# Training for Wellness in Pediatric Oncology: A Focus on Education and Hidden Curricula

**DOI:** 10.3390/curroncol29080440

**Published:** 2022-08-04

**Authors:** Fyeza Hasan, Reena Pabari, Marta Wilejto

**Affiliations:** 1 Division of Haematology/Oncology, Department of Paediatrics, The Hospital for Sick Children, Toronto, ON M5G 1X8, Canada; 2 Division of Hematology/Oncology, Department of Pediatrics, London Health Sciences Center, London, ON N6A 5W9, Canada

**Keywords:** oncology, pediatric, palliative, psychosocial, wellness, burnout, education, hidden curriculum

## Abstract

Pediatric oncologists have the privilege of caring for children and families facing serious, often life-threatening, illnesses. Providing this care is emotionally demanding and associated with significant risks of stress and burnout for oncologists. Traditional approaches to physician burnout and wellbeing have not emphasized the potential roles of education and training in mitigating this stress. In this commentary, we discuss the contribution that education, particularly in the areas of palliative and psychosocial oncology, can make in preparing oncologists for the work that they do. We argue that by adequately providing oncologists with the skills they need for their work, we can reduce their risk of burning out. We also discuss the importance of paying attention to hidden and formal curricula to ensure that messages provided in formal education programs are supported by informal training experiences.

## 1. The Weight of Pediatric Oncology

In our (probably biased) opinion, being a pediatric oncologist provides truly the best of medicine: the nature of the conditions we treat and the care we provide means that we see our patients frequently, and over many years. In doing so, we have the honor of building close, long-term relationships with children and their families, accompanying them through some of the hardest moments of their lives. As oncologists, we work with families under significant stress, helping them to navigate complex medical situations and caring for children who face the risk of serious complications, including death. While we feel privileged to do this important work, we acknowledge that the intensity of this type of care can have a personal cost.

Previous studies demonstrate the impact of this emotionally challenging work on care providers. Faced with the death of a patient, pediatric oncologists describe experiencing feelings of guilt, personal failure, self-doubt, and helplessness [1,2]. Additionally, care providers report feeling disconnected from their usual support networks of family and friends, who are often unable to relate to the challenges of caring for children with cancer. The combination of managing difficult situations with a lack of support contributes to the serious risk of burnout among this group of caregivers. Although there is a paucity of data on the incidence of burnout in pediatric oncologists, burnout is astonishingly reported to affect 35–60% of medical oncologists [3].

Burnout has been defined as a combination of depersonalization, emotional exhaustion and a reduced sense of personal accomplishment [4]. It is not a new concept in the medical field, and has been a subject of increasing interest in recent years. Several studies have attempted to identify risk factors for burnout. Factors associated with burnout in oncologists include demographic factors such as being younger, or being single, personal factors such as having poor physical or psychological health, and workplace related-factors including having a high workload and inadequate work–life balance [5]. However, for many of these factors, it is unclear whether they are causes or consequences of burnout. Additionally, while it can be helpful to use these risk factors to identify individuals at risk of burning out, it is not always possible for individuals to address these risk factors. Perhaps as a result, research on burnout has shifted beyond individual factors to exploring the culture in which burnout occurs. Ariely et al. identified key contributing factors within medical culture that may lead to burnout, including asymmetrical rewards, loss of autonomy, and cognitive scarcity [6]. They define asymmetrical rewards as an aspect of a culture that demands perfection, does not celebrate success and penalizes errors heavily. Loss of autonomy arises from physicians’ lack of control over how their time and energy is spent. Physicians face multiple competing demands on their time and the expectation of 24-h engagement, leading to cognitive scarcity. Cognitive scarcity results from the inherent conflict between physician goals; namely to provide the best possible care for patients, and the structure of the healthcare system in which they work, which limits the time available to do so.

## 2. The Impact on Trainees

The years of residency and fellowship training are a formative period in a physician’s career. They are also a time when physicians may be most affected by the aforementioned cultural factors that contribute to burnout. It is surprising, then, that burnout in trainee physicians and factors that support trainee wellbeing have received less attention in the literature when compared to staff physicians. Nonetheless, there is growing evidence to demonstrate that medical trainees are at a high risk of depression and burnout, particularly those in residency training [7,8,9,10]. A recently published meta-analysis identified key factors contributing to trainee burnout [11]. Of note, trainees’ concerns about the quality of patient care and poor patient outcomes were frequently identified as contributing to burnout and stress.

As in other specialties, trainees in pediatric oncology are vulnerable to the risks of burnout. We know that the experiences of trainees in treating children at the end of life are particularly complex. This relates in part to not being part of the patient’s primary team, but forming relationships with families during routine care. When children subsequently die, trainees who may have moved on to other rotations or may be off-work following overnight call, may not hear of deaths, preventing them from attending funerals or having closure with a family. Their ability to cope with these emotionally intense situations is limited by their inexperience in dealing with grief and the general vulnerability that trainees feel in training programs where their actions and reactions are subject to judgment and scrutiny [12]. Inadequate training experience has also been associated with measures of burnout once oncologists enter independent practice further strengthening the need to intervene early [13,14]. 

Recognizing burnout among health care providers is important, as it can have far-reaching consequences for both patients and physicians, leading to high rates of physician substance abuse, depression, suicidal ideation and medical errors [15]. With respect to medical trainees, burnout is linked to poorer performance in clinical and communication scenarios, such as breaking bad news and lower patient satisfaction scores [16,17,18].

## 3. Traditional Approaches to Burnout and Wellness

In recent years medical schools and hospitals have developed strategies to improve trainee wellness and reduce burnout, including the implementation of duty hour restrictions and formal wellness programs. Wellness programs have traditionally incorporated a broad range of evidence-based approaches including mindfulness interventions, resilience training, stress management and exercise programs [19]. Studies have also shown that facilitated group sessions (Balint groups) and didactic curricula focusing on self-reflection and communication reduce stress and improve work satisfaction [20,21,22,23,24,25,26]. Despite the recent explosion in wellness interventions, the prevalence of burnout in trainees has remained largely unchanged over the last few decades, as noted in a recent systematic review [27]. The reasons for this are likely multifactorial, and may relate to unrecognized systemic factors ingrained in the culture of medicine. 

## 4. Shifting Our Focus

How can we cope with the emotional impact of our work as pediatric oncology trainees and physicians? While we believe in the value of traditional approaches to wellness as described above, we also believe that education can play an important role in preparing oncologists for their challenging work. Through education, individual trainees and physicians alike can be better prepared to navigate the complex situations surrounding childhood death and dying, in turn creating more resilient physicians with less risk of experiencing burnout. Furthermore, we believe that the medical education community also has the opportunity to create system-wide change by ensuring that the culture of medical training promotes resiliency and wellness. 

## 5. Educational Priorities and the Hidden Curriculum

Education for oncology trainees and physicians has historically focused on a limited range of topics, narrowly centered on the diagnosis and medical management of cancers, often at the expense of topics such as palliative care and psychosocial oncology. The World Health Organization defines palliative care as an ‘approach that improves the quality of life of patients and their families facing the problems associated with life-threatening illness, through the prevention and relief of suffering by means of early identification and impeccable assessment and treatment of pain and other problems, physical, psychosocial and spiritual’ [28]. In order to provide the high-quality palliative and psychosocial care that patients and their families require, it is clear that oncology trainees and physicians need dedicated training. Despite the need for this training, many organizations overseeing resident education have only recently recognized the need for palliative care education for pediatric oncology fellows. For example, the Royal College of Physicians and Surgeons of Canada only mandated palliative care education for pediatric oncology fellows in 2013 [29]. Despite this requirement, formal curricula are left up to the discretion of training programs. 

Our 2017 survey of Canadian fellows and their program directors showed that the provision of palliative care education for pediatric oncology fellows varies widely [30]. Many programs provided only a few hours of training per academic year and several important subjects, such as the management of patient, family and physician grief, were not covered universally. Despite rating psychosocial skills as being important to acquire, fellows’ comfort levels in managing psychosocial issues were low. Importantly, distress was commonly reported by trainees, who identified a lack of education and comfort in palliative care skills as contributing to this distress. This inadequate provision of palliative care and psychosocial oncology training in pediatric oncology is seen in other areas of medicine, including adult oncology [31,32,33,34,35,36,37,38]. 

Excluding a subject from a formal educational curriculum sends a message to trainees and physicians alike, that this subject is not central to the core responsibilities of their practice. In minimizing the time and attention paid to psychosocial and palliative care education within an oncology training curriculum, we provide a subtle indication that these aspects of care are peripheral to the ‘real’ work of oncology.

It is widely recognized that formal educational curricula are not the only sources of learning for medical trainees [39,40]. Formal training is supplemented by informal teaching that happens at the bedside, in meetings, in conversations that occur in hallways etc. This informal or hidden curriculum is typically sporadic, often unplanned, and highly idiosyncratic. Much of this informal curriculum may be delivered by role modeling of behaviors by senior trainees and staff, particularly in relation to the provision of psychosocial care. The informal curriculum is both highly dependent on and influences the medical culture in which training occurs. Again, by leaving an important area of education to a highly informal mode of teaching, we demonstrate its lack of perceived value. It is also important to acknowledge that while the informal or hidden curriculum may provide valuable learning, it may also contradict the teaching provided in the formal curriculum [41,42,43]. For example, the importance of palliative care may be formally taught, but dismissive comments from senior staff may undermine this teaching.

Education and training may also help oncology trainees and physicians learn how to monitor their own wellbeing and take action to protect it. Fisch proposed a model of buoyancy for oncology providers [44]. He describes buoyancy as being the opposite of burnout. According to Fisch’s model, several factors contribute to our wellbeing as oncologists. Some of these factors include: a sense of autonomy over your work, exercising skill, meaningful relationships, recognizing your fears and managing them, accepting loss and managing grief, and a sense of purpose. By being aware of these factors, and self-monitoring how we are doing in relation to these factors, Fisch suggests that we can maintain our buoyancy or resiliency against burnout. As educators, we must maintain awareness of how the hidden curriculum impacts oncologists’ perceptions of the value of monitoring and addressing such factors. By doing so, we ensure that the importance of this strategy in physician wellness is not diminished.

## 6. Conclusions

Being surrounded by childhood death and dying clearly has a profound impact on the wellbeing of pediatric oncologists and trainees alike. Inadequate training not only has implications for the quality of care provided to children with cancer and their families but also for the wellness of those tasked with providing that care. Being inadequately prepared to navigate challenging patient care scenarios has been associated with trainee distress and may contribute to burnout. The risk of burnout is further compounded by a medical culture that doesn’t always support wellness. Palliative care and psychosocial aspects of oncology should be formally included in education programs to provide trainees with the skills needed to confidently approach the challenges they will inevitably face. Attention should also be paid to the informal and hidden curricula to ensure consistent messaging about the importance of palliative and psychosocial care as well as taking care of trainee and physician wellbeing. By providing high-quality palliative and psychosocial oncology training, integrated into oncology teaching programs and routine oncology practice, we can improve the care we provide to our patients and families while reducing the risk of burnout in care providers. If pediatric oncology truly is the best of medicine, we owe it to ourselves and our patients to prepare as best we can for the work we are honored to do. 

## Data Availability

Not applicable.
